# HIV treatment and monitoring patterns in routine practice: a multi-country retrospective chart review of patient care

**DOI:** 10.12688/f1000research.15169.3

**Published:** 2019-01-04

**Authors:** Baba M Musa, Everistus Ibekwe, Stanley Mwale, Daniel Eurien, Catherine Oldenburg, Gary Chung, Richard F Heller

**Affiliations:** 1 Department of Medicine, Aminu Kano Teaching Hospital, Bayero University Kano, Kano, Nigeria; 2 Faculty of Health, Psychology and Social Care, Manchester Metropolitan University, Manchester, M15 6GX, UK; 3 Centre for Infectious Disease Research in Zambia, Lusaka, 10101, Zambia; 4 Advanced Field Epidemiology Training Program , Kampala, Uganda; 5 The Francis I. Proctor Foundation for Research in Ophthalmology, University of California, San Francisco, San Francisco, CA, 94143, USA; 6 Department of Ophthalmology, University of California, San Francisco, San Francisco, CA, 94143, USA; 7 Johnson & Johnson, New Brunswick, NJ, 08901, USA; 8 People’s Open Access Education Initiative, Manchester, M30 9ED, UK

**Keywords:** HIV, quality of care, variation, Public Health

## Abstract

**Background:** A study of patient records in four HIV clinics in three sub-Saharan African countries examined routine clinical care patterns and variations.

**Methods: **Clinic characteristics were described, and patient data extracted from a sample of medical records. Data on treatment, CD4 count and viral load (VL) were obtained for the last visit in the records, dates mainly between 2015 and 2017, patient demographic data were obtained from the first clinic visit.

**Results**: Four clinics, two in Nigeria, one in Zambia and one in Uganda, all public facilities, using national HIV treatment guidelines were included. Numbers of patients and health professionals varied, with some variation in stated frequency of testing for CD4 count and VL. Clinical guidelines were available in each clinic, and most drugs were available free to patients. The proportion of patients with a CD4 count in the records varied from 84 to 100 percent, the latest median count varied from 269 to 593 between clinics. 35% had a record of a VL test, varying from 1% to 63% of patients. Lamivudine (3TC) was recorded for more than 90% of patients in each clinic, and although there was variation between clinics in the choice of antiretroviral therapy (ART), the majority were on first line drugs consistent with guidelines.  Only about 2% of the patients were on second-line ARTs. In two clinics, 100% and 99% of patients were prescribed co-trimoxazole, compared with 7% and no patients in the two other clinics.

**Conclusions: **The wide variation in available clinic health work force, levels and frequency of CD4 counts, and VL assessment and treatment indicate sub-optimal adherence to current guidelines in routine clinical care. There is room for further work to understand the reasons for this variation, and to standardise record keeping and routine care of HIV positive patients.

## Introduction

In addition to choice of appropriate drugs, best practice management of patients with HIV requires monitoring response to treatment and disease progression. This includes tracking clinical immunological, and virological data on patients at diagnosis and on follow-up. There is a rich literature guiding HIV treatment, and guidelines are developed and updated regularly as new evidence comes to light ^1– 5^ (see http://www.who.int/hiv/pub/guidelines/en/ for an historic list of guidelines produced by the World Health Organisation). The implementation of standardized protocols for treatment and investigations may vary in resource constrained countries, with differences in resources available for such services. For example, the current guidelines for antiretroviral therapy (ART) requires all patients on ART to have viral load test at six months and 12 months ^5^, implementation might vary based on availability of resources to support viral load testing. While a number of reports of current treatment and testing practice are available from resource-limited settings, these are mainly in the context of patients enrolled in research centres or in research studies ^6– 8^. Such settings often have substantially more resources than routine clinical practices, and as such practice patterns may differ from routine practice, where issues such as clinic and patient resources or drug stock-outs can affect care. This study was designed to explore variation in the evaluation, treatment, care and follow-up among patients diagnosed with HIV in routine care settings in low- to middle-income countries.

## Methods

Interest in participating in this study was sought among the Masters of Public Health Alumni of Peoples-uni ^9^. There was no prior determination of the numbers of centres required or their geographical setting. A protocol was developed by those who responded ( Supplementary File 1), which included the Research questions as follows: 1. Among a group of patients diagnosed with HIV by a health care facility newly diagnosed between 2 and 3 years previously, what proportion have standards of care in terms of the tests and treatments they receive and follow up to 2 years after diagnosis documented in their records? 2. Among the health care facilities in which the above patients are cared, what treatment, testing and referral facilities are available? 3. What is the extent of the variation in the above measures between facilities and countries, as well as in the availability of appropriate evidence based practice guidelines? A retrospective patient cohort was created via standardized record review, and clinic characteristics determined through an online survey form.

A data collection instrument was developed, based on previous research and publications and management guidelines ^1– 5^. A spreadsheet was created with coding instructions ( Supplementary File 2). In addition, data on the characteristics of the setting for each facility were collected in early 2017 by each investigator in consultation with local clinic staff, and entered onto an online survey form ( Supplementary File 3). This included country and city, hospital or other healthcare facility, number of patients seen, and what diagnostic, treatment, referral and follow-up facilities are available. Clinic and patient data were de-identified to maintain confidentiality. Four clinics from three countries in sub-Saharan Africa chose to participate in the study.

For clinic BA, a public hospital clinic in northern Nigeria, patients were selected in sequence of attendance as new patients, starting January 1, 2013 for whom records were also available over the next two years. Patient demographics were those obtained at the first visit, and CD4 count and ART treatments were recorded at each visit, with analysis relating to data recorded at the most recent visit.

For clinic EV, a public hospital clinic also in Nigeria, all existing patients were re-tested with an ELISA method in 2013/14, and patients were randomly selected for this study among those who tested positive at that time. CD4 count and treatments recorded were those at the latest visit, and patient demographics were those in the records from their first visit.

For clinic MW, a public community-based clinic in Zambia, patients were randomly selected from those present on the patient registry in 2015/16. At that time, patients were reviewed for the need to start on highly active antiretroviral therapy (HAART) based on CD4 count, and the ART regime recorded was that started as a result of that review at that time. Data on patient demographics were those present in the records, often this would be prior to the date of entry to this study.

For clinic AM, a public hospital based clinic in Uganda, the sampling frame was the register of all clients on active ART, and every third patient file was retrieved from files ordered according to clinic appointment date. The CD4 counts and ART regimes recorded were for the most recent measures in the records, and patient demographics were those in the records from their first visit.

### Measurement of study factors

Data on individual patients were extracted from medical records, including age, gender, and baseline clinical data at diagnosis and follow-up. Clinical information included clinical, immunological and virological information on patients at diagnosis and on follow-up, prescription of ART and other drug choices. Potentially important co-infections and/or co-morbidities, identified from the literature, were coded as indicated in Supplementary File 2.

### Measurement of outcome factors

Details of the tests ordered and their results, and treatments ordered were extracted from medical records. Individual patients were not contacted. Data were obtained from the records, and no attempt was made to validate the information. Missing data were recorded as missing, and not explored further.

A pilot study tested the feasibility of the data collection and the method of recording on to a spreadsheet. Data were collected by a research assistant in each setting, using the spreadsheet, from examination of individual records.

### Ethics requirements

As a retrospective patient records study, consent was not requested from individual patients. Ethics approval was sought and obtained in each setting from the appropriate authority (see Ethical approval and consent section). Considerable care was taken not to reveal the identity of any individual and all data were de-identified. The spreadsheet for the recording of data only had an identification number, and the key to the identity of each patient was kept separately to maintain confidentiality. To further this, the clinics names and exact locations have been removed.

### Sample size considerations

Each centre was asked to obtain information from at least 100 patients.

### Data analysis

Descriptive statistics were used to characterise the study population. Data distributions were assessed, checking for skewness and kurtosis. Data summary statistics were generated. Categorical variables were summarized using proportions while continuous variables were summarized using medians and interquartile ranges. Due to differences in data extraction across sites, no statistical analyses comparing across sites was conducted. All analyses were conducted in Stata 14.1 (StataCorp, College Station, X) and R version 3.3.0 (The R Foundation for Statistical Computing).

## Results

Ten alumni expressed interest in participating, three centres were able to pilot the data collection instrument and four centres in three countries participated in the data collection for the study. Overall, data were abstracted for 600 patients.

Table 1 shows the characteristics of the 4 clinics. The number of patients seen per month varied from 500 to 4200, and the number of doctors, nurses, and allied health professionals available at the clinics varied from 1 to 50, 10 to 65, and 6 to 45 respectively. There was some variation in the stated frequency of testing for CD4 count (either every three or six months) and for measurement of viral load. Each clinic had availability of clinical guidelines, and most drugs were available free of charge to patients. All clinics have access to ART and co-trimoxazole.

**Table 1.** Characteristics of the 4 participating clinics.

	Nigeria 1 [BA]	Nigeria 2 [EV]	Zambia [MW]	Uganda [AM]
Hospital or community	Hospital	Hospital	Community	Hospital
Public or private	Public	Public	Public	Public
HIV screening	Separate clinic	In clinic	In clinic	In clinic
Number of patients per month	500	4200	1200	1200
Number of doctors	6	50	1	2
Number of nurses	10	65	15	10
Number of allied health professionals	21	45	6	6
Access to HIV clinical guidelines	Yes, National, Electronic	Yes, National, Electronic and paper	Yes, National, Paper	Yes, National, Electronic and paper
CD4 count	Yes, each 3 months	Yes, each 3 months	In central lab, each 6 months	Yes, transported to lab, each 6 months
Viral load	Yes, each year	Yes, on referral, occasionally	Yes, on referral, when indicated	Yes, each 6 months for adolescents, yearly for adults
Drugs available free	All ART, Co-trimoxazole	All	All (IDV and ATV not free)	All
CD4 to start treatment	<350	<350	<350	<350
Referral	Yes, TB	Yes, complicated eg multi-drug resistance	Yes, if fail treatment	Yes, patients with other medical conditions

ART antiretroviral therapy

IDV indinavir

ATV atazanavir

**Figure 1.**
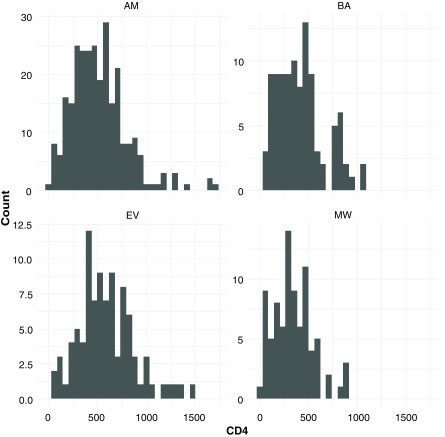
Distribution of CD4 counts in each of the four clinics.

Table 2 shows the patient characteristics and test results. Age, gender and body mass index (BMI) were similar between clinics, with overall median age of 31 years, 62% were female and median BMI was 20. The proportion of patients with a CD4 count in the records varied from 84 to 100 percent, and the latest median count varied from 269 to 593 between the clinics. 209 (35%) had a record of a viral load test with 81% of then having a viral load of less than 50 copies/µL. Only 23 (19%) of 119 patients had records of AIDS defining illnesses. Figure 1 shows the variation in the distribution of the CD4 counts in each of the four clinics. As reported in the records, 58% of the latest CD4 counts were from 2016 or 2017, 27% from 2015, and the remaining 15% earlier; 95% of the viral load tests were from 2016 or 2017. There was no information about the timing of the reports being given to the patients themselves.

**Table 2.** Patient characteristics.

	BA (N=100)	EV (N=100)	MW (N=100)	AM (N=300)	Overall (N=600)
Age (median, IQR)	32 (28 to 40)	28 (21 to 37)	33 (29 to 39)	31 (23.5 to 38)	31 (24 to 38)
Female sex	57 (57%)	62 (62%)	55 (55%)	197 (66%)	371 (62%)
BMI at presentation ^1^	22.8 (19.5 to 31.2)	No data	19.5 (17.9 to 21.8)	20.0 (18.3 to 22.2)	20.0 (18.2 to 22.3)
Number of patients w/CD4 count data	100	94 (94%)	84 (84%)	279 (93%)	558 (93%)
Latest CD4 Count (median, IQR)	269 (177 to 461)	593 (390 to 880)	307 (169 to 471)	499 (321 to 691)	436 (267 to 471)
Proportion of patients w/viral load	14 (14%)	6 (6%)	1 (1%)	188 (63%)	209 (35%)
Latest viral load <50 50-9,999 10,000-99,999 >99,999	6 (43%) 1 (7%) 3 (21%) 4 (29%)	2 (33%) 4 (67%) 0 0	1 (100%) 0 0 0	160 (85%) 0 28 (15%) 0	169 (81%) 5 (2%) 31 (15%) 4 (2%)
AIDS defining illness	2/2(100%)	7/21(33%)	14/96(15%)	No data	23/119(19%)

^1^Missing BMI data: AM: 3/300, BA: 81/100, EV: 100/100, MW: 14/100 (total 198/600)

Table 3 shows the treatment regimes. Lamivudine (3TC) was recorded for more than 90% of patients in each clinic, but otherwise there was considerable variation between clinics in the choice of ART. Tenofovir disoproxil fumarate (TDF) was prescribed for 317 (53.9%) of studied patients. Efavirenz (EFV) was prescribed for 326 (55%) of the patients, making combination of TDF/3TC/EFV the most common ART combination used by studied patients. In two clinics, 100% and 99% of patients were prescribed co-trimoxazole prophlaxis, compared with 7% and no patients in the two other clinics. There were 6 patients on lopinavir and 8 on atazanavir (second line treatment choices).

**Table 3.** Antiretroviral therapy (ART) Regimens.

ART regimen contains	BA (N=100)	EVT (N=94)	MW (N=99)	AM (N=295)	Overall (N=588)
AZT zidovudine TDF tenofovir disoproxil fumarate FTC emtricitabine 3TC lamivudine NVP nevirapine IDV indinavir RTV ritonavir d4T stavudine ABC abacavir LPV lopinavir ATV atazanavir EFV efavirenz	28 (28.0%) 70 (70.0%) 10 (10.0%) 90 (90.0%) 24 (24.0%) 0 0 0 2 (2.0%) 3 (3.0%) 6 (6.0%) 69 (69.0%)	87 (92.6%) 3 (3.2%) 0 94 (100%) 91 (96.8%) 0 0 0 0 0 1 (1.1%) 2 (2.1%)	0 90 (90.9%) 5 (5.2%) 94 (95.0%) 0 0 0 6 (6.4%) 9 (9.1%) 3 (3.0%) 0 93 (93.9%)	113 (38.3%) 154 (52.2%) 0 276 (93.6%) 111 (37.6%) 0 0 0 9 (3.1%) 0 1 (0.3%) 162 (54.9%)	228 (38.8%) 317 (53.9%) 15 (2.6%) 554 (94.2%) 226 (38.4%) 0 0 6 (1.0%) 20 (3.4%) 6 (1.0%) 8 (1.4%) 326 (55.4%)
Co-trimoxazole	100(100%)	7 (7.5%)	0	292 (98.9%)	399 (66.6%)

De-identified data collected from clinical records used to create Tables 2 and 3 and Figure 1 Scheme used to code the data is available as Supplementary File 2
Copyright: © 2019 Musa BM et al. 2019 Data associated with the article are available under the terms of the Creative Commons Zero "No rights reserved" data waiver (CC0 1.0 Public domain dedication).

## Discussion

Data were collected according to the standardised data collection instrument ( Supplementary File 2), with data on treatment regimes and CD4 counts relating to the latest information in the records (dates mainly between 2015 and 2017). While we initially planned to collect data on a cohort of patients enrolled two years before data collection in each clinic, this did not prove feasible and patients had presented to the clinics at variable times.

This study found modest documentation of clinical activities, with a wide variation in available clinic health work force, frequency of CD4 counts and levels and viral load assessment. The two clinics in Nigeria had markedly different caseloads. The centre with the high caseload is one of the oldest in Nigeria and provides incentives to this population of patients (free medical care to family members, distribution of food supplements and sometimes fares, etc.), Differences in case load may reflect population distribution and heterogeneity in HIV prevalence, or that patients may seek care far away from their base to avoid stigmatization. Variation in case load can lead to differences in efficiency and quality of care, and potentially could have implications for care outcomes. However determining its impact on outcome would a require a properly powered longitudinal study.

While most patients had a CD4 count in the records, there was a wide spread of latest CD4 counts, with both within- and between-centre variation in the CD4 counts. Only one in three patients had a record of viral load at any time during the course of treatment, and most of these had an undetectable level (<50 copies/µL). Some of the absence of viral load might be poor record keeping, and the high proportion of undetectable results might reflect an absent rather than a low test result, and hence not an indicator of treatment success. Over the years the CD4 threshold for commencing ART had been lowered, as can be seen in Table 4, with the most current guideline requiring a “test and treat approach” ie eliminating CD4 threshold as a prerequisite for commencement of ART ^5^. While this guideline is based on sound evidence, it adds an unanticipated number of potential eligible patients for ART care, with attendant strain on countries with an already fragile economy. The low frequency of viral load test results might also reflect resource limitations.

**Table 4.** Summary of key features of World Health Organisation guidelines.

When to start ART in adults		
TIME	REFERENCE	RECOMMENDATION
2002	Scaling up antiretroviral therapy in resource-limited settings: guidelines for a public health approach ^1^ http://www.who.int/hiv/pub/guidelines/pub18/en/	ART treatment if: • CD4 <200 cells/mm3 WHO stage I, II, or III OR • WHO Stage IV AIDS-defining illness, irrespective of CD4 count • First line therapy ZDV/3TC/EFZ or ZDV/3TC/NVP
2006	Antiretroviral therapy for HIV infection in adults and adolescents. Recommendations for a public health approach (2006 revision) ^2^ http://www.who.int/hiv/pub/arv/adult/en/	• All adolescents and adults including pregnant women with HIV infection and CD4 counts of ≤200 cells/mm3, should start ART, • First line treatment NRTI AZT or TDF combined with either 3TC or FTC; NNRTI, either EFV or NVP, should be added
2010	Antiretroviral therapy for HIV infection in adults and adolescents. Recommendations for a public health approach: 2010 revision ^3^ http://www.who.int/hiv/pub/arv/adult2010/en/	• All adolescents and adults including pregnant women with HIV infection and CD4 counts of ≤350 cells/mm3, should start ART, • First-line therapy should consist of an NNRTI + two NRTIs, one of which should be AZT or TDF • Second-line ART should consist of a ritonavir-boosted protease inhibitor (PI) plus two NRTIs, one of which should be AZT or TDF • Irrespective of CD4 cell counts, patients coinfected with HIV and TB should be started on ART • Irrespective of CD4 cell counts or WHO clinical stage, patients who require treatment for HBV infection should start ART.
2013	Consolidated guidelines on the use of antiretroviral drugs for treating and preventing HIV infection. Recommendations for a public health approach ^4^ http://www.who.int/hiv/pub/guidelines/arv2013/download/en/	• As a priority, ART should be initiated in all individuals with severe or advanced HIV clinical disease (WHO clinical stage 3 or 4) and individuals with CD4 count ≤350 cells/mm3 • ART should be initiated in all individuals with HIV with CD4 count >350 cells/mm³ and ≤500 cells/mm3 regardless of WHO clinical stage • ART should be initiated in all individuals with HIV regardless of WHO clinical stage or CD4 cell count in the following situations: • Individuals with HIV and active TB disease (strong recommendation, low-quality evidence). • Individuals coinfected with HIV and HBV with evidence of severe chronic liver disease • Partners with HIV in serodiscordant couples should be offered ART to reduce HIV transmission to uninfected partners (strong
2015/6	Consolidated guidelines on the use of antiretroviral drugs for treating and preventing HIV infection. Recommendations for a public health approach - Second edition ^5^. http://www.who.int/hiv/pub/arv/arv-2016/en/	• Retesting prior to enrollment in care ART should be initiated in all adults living with HIV, regardless of WHO clinical stage and at any CD4 cell count • As a priority, ART should be initiated in all adults with severe or advanced HIV clinical disease (WHO clinical stage 3 or 4) and adults with a CD4 count ≤350 cells/mm3 • ART should be started in all TB patients living with HIV regardless of CD4 count • Preferred first line ART regimen TDF + 3TC (or FTC) + EFV • Combinations of ATV and LPV are the preferred boosted PI options for second-line ART • Routine viral load monitoring can be carried out at 6 months, at 12 months and then every 12 months thereafter if the patient is stable on ART to synchronize with routine monitoring and evaluation reporting • CD4 count every 6 months until in settings where routine viral load monitoring is available, until are stable on ART • Viral load is recommended as the preferred monitoring approach to diagnose and confirm treatment failure

ART antiretroviral therapy AZT zidovudine : ZDV retrovir 3TC lamivudine EFV (EFZ) efavirenz NVP nevirapine NRTI Nucleotide analog reverse transcriptase inhibitor TDF tenofovir disoproxil fumarate FTC emtricitabine NNRTI Non-nucleoside reverse transcriptase inhibitor HBV Hepatitis B virus LPV lopinavir

Most patients were on TDF/3TC/EFV drug regimen, which is consistent with current guideline advice for first line treatment. Only 2% of the patients were on second-line ART, varying from 9% to less than 1% between clinics. It is likely that most of the studied patients are still on a potent first line ART judging by the low second-line ART usage. Use of a regimen with a low pill burden would enhance therapy adherence and might lead to reduced need for switching patients to second line ART ^10^. TDF/3TC/EFV drug regimens have a low pill burden, and may contribute to the low usage of second-line ART regimens.

The variation in the use of co-trimoxazole, from almost universal in two clinics, to negligible in the other two clinics, is an extreme example of the variation we identified. The reason for this variation despite the guideline recommendation is not clear, but it is possible that it is a result of local clinic policy settings, possibly related to concerns about increasing antibiotic resistance. Variation in the use of co-trimoxazole has been found in other resource-limited settings, while efforts are being made to improve the rates of use ^11^.

While each clinic reported having access to treatment guidelines, those current at the time of the data collection do not appear to have been universally followed. The guidelines also do change regularly, as shown in Table 4. For example in relation to the frequency of CD4 count monitoring and the CD4 count threshold at which treatment should be started have changed since the time relating to the study data - new guidelines recommend starting treatment regardless of the CD4 count ^5, 12^. Some discrepancy between WHO guidelines and actual implementation in practice may arise from the time it takes to implement new guidelines, or due to lack of resources to immediately initiate all patients with HIV on ART. We see considerable between-clinic variation in a number of key management strategies reported by the clinics, from the recording of CD4 counts, median CD4 counts on treatment, and treatments used. This variation is consistent with a previous survey of stated management practices in 6 sub-Saharan countries ^13, 14^.

The management of patients in routine clinical care has been shown to differ from that seen in clinical trials ^15^, as well as to lead to worse clinical outcomes, although it is beyond the scope of our study to explore outcomes. Findings of variation from standard management practice has previously been reported from routine care settings in other parts of sub-Saharan Africa including Ethiopia ^16^, Uganda ^17^ and Tanzania ^18^, mostly in single isolated centres. Here we present combined data on health system related measures across multiple sub-Saharan African HIV treatment sites. Hence, this study adds to the literature a current examination of the routine care provided to patients with HIV, rather than that in the context of a clinical trial. The study adds information from a number of centres in multiple countries, using a common protocol, of both the characteristics of the clinics and of the care given in these clinics.

In the absence of standardised record keeping systems, it is difficult to make clear comparisons of management and outcome in routine clinical care. Our findings suggest that there is room for further work to understand the reasons for these record gaps, and to standardise the record keeping in routine care of HIV positive patients ^19– 21^. The potential of electronic medical records to improve records could be explored ^22^.

Our study has a number of limitations. First, this study was an analysis of data extracted from existing medical records, which are prone to error including missing data. We have found missing data where it should ordinarily not be missing. We cannot comment on other factors that may have contributed to missing data, such as whether tests were not done or if they were not recorded. A future study should perform an audit of the medical records to determine the reason for missing data and hence the potential effect on the study findings. Second, some of the variation between centres may be due to differences in patient populations which we were unable to capture, even though we selected clinics involved in routine clinical care. Third, since this is a descriptive study and not hypothesis testing, it was not feasible to determine sample size in advance. A pragmatic approach was adopted, with each centre expected to obtain information from the first 100 patients presenting over a 6-month period, or from at least 100 patients. Fourth, participation in the study was restricted to few countries, and to individual clinics, which may not be representative of the national picture in these countries. A prospective cohort study on representative samples would provide a more robust study design with more detailed quantitative data to delineate care dynamics and to provide longitudinal data to better understand how clinical practice in these settings is linked to patient outcomes.

## Conclusions

We demonstrate a wide variability in compliance with HIV treatment guidelines in four routine care settings in sub-Saharan Africa, as well as gaps in the records available. The findings of this study may provide an explanation for heterogeneous HIV treatment outcomes across sub-Saharan Africa. In spite of the limitations, these data underscore the need for an in-depth study to address compliance with HIV treatment guidelines and best practice. While electronic medical record implementation might be a challenge for many HIV care points in sub-Saharan Africa, our findings emphasize the need for more robust interim paper-based medical record keeping.

## Ethics approval and consent

Ethics approval was sought in each setting from the appropriate authority. For two of the centres, research ethics committees gave approval. In two of the centres, the ethics committees stated that they did not require formal approval from them, however approval to access records was obtained from the relevant District Health Office/r. Details for each clinic as follows:

Clinic BA: Ethics approval obtained from Aminu Kano Teaching Hospital Research Ethics Committee.

Clinic EV: Ethics approval obtained from Research and Ethics Committee of State House Medical Centre, Abuja.

Clinic MW: University of Zambia Biomedical Research Ethics Committee contacted and advised to notify the Lusaka District Health Office (LDHO) who gave approval.

Clinic AM: Eastern Uganda AIDS Support Organization (TASO) contacted who recommended no need for an approval but rather write to the Amuria District Health Officer (DHO) for permission to have access to the hospital records, which was given.

Informed consent was not obtained from individual patients since this was a records study with appropriate institutional approval, strict confidentiality arrangements, and no patient contact, as stated in the manuscript.

## Data availability

The data referenced by this article are under copyright with the following copyright statement: Copyright: © 2019 Musa BM et al.

Data associated with the article are available under the terms of the Creative Commons Zero "No rights reserved" data waiver (CC0 1.0 Public domain dedication). 

Dataset 1. De-identified data collected from clinical records used to create Table 2 and Table 3 and Figure 1. Scheme used to code the data is available as Supplementary File 2. 10.5256/f1000research.15169.d206333 ^23^

All data underlying the survey responses from clinic informants for Table 1 are available as part of the article.

## Supplementary material

Supplementary File 1 – Study protocol

Supplementary File 2 – Data coding scheme

Supplementary File 3 – Written from of online survey

## References

[1] World Health Organisation: Scaling up antiretroviral therapy in resource-limited settings: guidelines for a public health approach.2002 Reference Source 12154788

[2] World Health Organisation: Antiretroviral therapy for HIV infection in adults and adolescents: Recommendations for a public health approach (2006 revision).2006 Reference Source 23741771

[3] World Health Organisation: Antiretroviral therapy for HIV infection in adults and adolescents: Recommendations for a public health approach: 2010 revision. Reference Source 23741771

[4] World Health Organisation: Consolidated Guidelines on the Use of Antiretroviral Drugs for Treating and Preventing HIV Infection: Recommendations for a Public Health Approach.2013. 24716260

[5] World Health Organisation: Consolidated Guidelines on the Use of Antiretroviral Drugs for Treating and Preventing HIV Infection: Recommendations for a Public Health Approach. Second edition.2016. 27466667

[6] Petersen M Balzer L Kwarsiima D: SEARCH test and treat study in Uganda and Kenya exceeds the UNAIDS 90-90-90 cascade target by achieving 81% population-level viral suppression after 2 years. *AIDS* 2016; Durban, South Africa. Reference Source

[7] Ayele TA Worku A Kebede Y: Choice of initial antiretroviral drugs and treatment outcomes among HIV-infected patients in sub-Saharan Africa: systematic review and meta-analysis of observational studies. *Syst Rev.* 2017;6(1):173. 10.1186/s13643-017-0567-7 28841912 PMC5574138

[8] Slaymaker E McLean E Wringe A: The Network for Analysing Longitudinal Population-based HIV/AIDS data on Africa (ALPHA): Data on mortality, by HIV status and stage on the HIV care continuum, among the general population in seven longitudinal studies between 1989 and 2014 [version 1; referees: 2 approved, 1 approved with reservations]. *Gates Open Res.* 2017;1:4. 10.12688/gatesopenres.12753.1 29528045 PMC5841576

[9] Heller RF Machingura PI Musa BM: Mobilising the alumni of a Master of Public Health degree to build research and development capacity in low- and middle-income settings: The Peoples-uni. *Health Res Policy Syst.* 2015;13:71. 10.1186/s12961-015-0064-1 26621526 PMC4665818

[10] Sahay S Reddy KS Dhayarkar S: Optimizing adherence to antiretroviral therapy. *Indian J Med Res.* 2011;134(6):835–849. 10.4103/0971-5916.92629 22310817 PMC3284093

[11] Bardfield J Agins B Palumbo M: Improving rates of cotrimoxazole prophylaxis in resource-limited settings: implementation of a quality improvement approach. *Int J Qual Health Care.* 2014;26(6):613–22. 10.1093/intqhc/mzu085 25335758

[12] Abdool Karim SS: Overcoming Impediments to Global Implementation of Early Antiretroviral Therapy. *N Engl J Med.* 2015;373(9):875–876. 10.1056/NEJMe1508527 26193047

[13] Church K Machiyama K Todd J: Identifying gaps in HIV service delivery across the diagnosis-to-treatment cascade: findings from health facility surveys in six sub-Saharan countries. *J Int AIDS Soc.* 2017;20(1):21188. 10.7448/IAS.20.1.21188 28364566 PMC5461119

[14] Ambia J Renju J Wringe A: From policy to practice: exploring the implementation of antiretroviral therapy access and retention policies between 2013 and 2016 in six sub-Saharan African countries. *BMC Health Serv Res.* 2017;17(1):758. 10.1186/s12913-017-2678-1 29162065 PMC5698969

[15] López-Martínez A O'Brien NM Caro-Vega Y: Different baseline characteristics and different outcomes of HIV-infected patients receiving HAART through clinical trials compared with routine care in Mexico. *J Acquir Immune Defic Syndr.* 2012;59(2):155–60. 10.1097/QAI.0b013e31823ff035 22107816

[16] Alemayehu YK Bushen OY Muluneh AT: Evaluation of HIV/AIDS clinical care quality: the case of a referral hospital in North West Ethiopia. *Int J Qual Health Care.* 2009;21(5):356–62. 10.1093/intqhc/mzp030 19684032 PMC2742392

[17] Burua A Nuwaha F Waiswa P: Adherence to standards of quality HIV/AIDS care and antiretroviral therapy in the West Nile Region of Uganda. *BMC Health Serv Res.* 2014;14:521. 10.1186/s12913-014-0521-5 25399661 PMC4239403

[18] Mapunjo S Urassa DP: Quality standards in provision of facility based HIV care and treatment: a case study from Dar es Salaam Region, Tanzania. *East Afr J Public Health.* 2007;4(1):12–8. 17907755

[19] Munthali T Musonda P Mee P: Underutilisation of routinely collected data in the HIV programme in Zambia: a review of quantitatively analysed peer-reviewed articles. *Health Res Policy Syst.* 2017;15(1):51. 10.1186/s12961-017-0221-9 28610616 PMC5470192

[20] Harklerode R Schawarcz S Hargreaves J: Feasibility of Establishing HIV Case-Based Surveillance to Measure Progress Along the Health Sector Cascade: Situational Assessments in Tanzania, South Africa, and Kenya. *JMIR Public Health Surveill.* 2017;3(3):e44. 10.2196/publichealth.7610 28694240 PMC5525003

[21] Hochgesang M Zamudio-Hass S Moran L: Scaling-up health information systems to improve HIV treatment: An assessment of initial patient monitoring systems in Mozambique. *J Med Inform.* 2017;97:322–330. 10.1016/j.ijmedinf.2016.11.002 27919390 PMC6056312

[22] Oluch T Kwaro D Ssempijja V: Better adherence to pre-antiretroviral therapy guidelines after implementing an electronic medical record system in rural Kenyan HIV clinics: a multicenter pre-post study. *Int J Infect Dis.* 2015;33:109–13. 10.1016/j.ijid.2014.06.004 25281905 PMC4987124

[23] Musa BM Ibekwe E Mwale S: Dataset 1 in: HIV treatment and monitoring patterns in routine practice: a multi-country retrospective chart review of patient care. *F1000Research.* 2018 10.5256/f1000research.15169.d206333 PMC6317496 30647906

