# Supplementary material for: HIV treatment and monitoring patterns in routine practice: a multi-country retrospective chart review of patient care
Source: F1000Res. 2019 Jan 4;7:713. Originally published 2018 Jun 8. [Version 3] doi: 10.12688/f1000research.15169.3 (PMC6317496; doi:10.12688/f1000research.15169.3)
Supplement: Supplementary file 4 [file f1000research-7-19421-s0003.tgz › 18618b0b-c421-42fb-91ac-26dace58e6ea.docx]

# HIV facilities survey

Alumni survey of HIV facilities

Thanks for taking part in this survey of HIV facilities

There are 26 questions in this survey

## Clinic characteristics

### []Country name *

Please choose only one of the following:

-
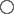
 Cameroon
-
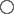
 Ghana
-
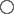
 India
-
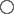
 Nepal
-
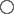
 Nigeria
-
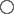
 Rwanda
-
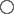
 Tanzania
-
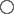
 Uganda
-
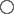
 Zambia
-
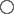
 Zimbabwe

### []Clinic name *

Please write your answer here:

### []

Hospital or community

### *

Please choose only one of the following:

-
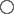
 Hospital
-
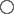
 Community
-
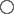
 Other

### []

Public or private

### *

Please choose only one of the following:

-
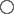
 Public
-
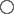
 Private
-
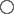
 Other

### []

Standalone clinic or part of ID clinic

### *

Please choose only one of the following:

-
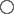
 Standalone clinic
-
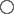
 Part of ID clinic
-
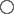
 Other

### []

Is HIV screening performed in the clinic?

### *

Please choose only one of the following:

-
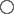
 HIV screening performed in the clinic
-
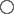
 HIV screening performed elsewhere
-
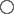
 HIV screening perormed in an HIV counseling and testing (HCT) center where the clinic has a formal linkage
-
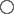
 Other

### []

Size of population served by the clinic

### *

Only numbers may be entered in this field.

Please write your answer here:


### []

Number of patients seen in the clinic in a month on average

### *

Only numbers may be entered in this field.

Please write your answer here:


### []

Number of doctors serving the clinic

Only numbers may be entered in this field.

Please write your answer here:


### []

Number of nurses serving the clinic

Only numbers may be entered in this field.

Please write your answer here:


### []

Number of allied health professionals serving the clinic

Only numbers may be entered in this field.

Please write your answer here:


## Questions for clinic doctor or manager

### []

Are you aware of the existence of clinical guidelines for HIV?

### *

Please choose only one of the following:

-
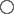
 Yes, I am aware of clinical guidelines for HIV, and I have access to them
-
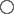
 Yes, I am aware of clinical guidelines for HIV, but I do not have access to them
-
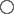
 No, I am not aware of clinical guidelines for HIV

### []

Is the guideline available to you national, regional or locally developed?

### *

Please choose only one of the following:

-
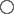
 The guideline is a national one
-
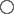
 The guideline is a regional one
-
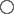
 The guideline is locally developed
-
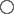
 I am not sure of the source of the guideline
-
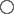
 I do not have access to a guideline
-
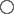
 Other

### []Is the guideline you use in paper or electronic form *

Please choose only one of the following:

-
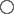
 Paper
-
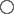
 Electronic
-
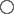
 Both paper and electronic
-
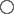
 I do not have access to a guideline
-
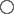
 Other

### []If you do have access to a guideline, can you give a reference to it?

Please write your answer here:

### []

Is a CD4 count available for patients in your clinic?

### *

Please choose only one of the following:

-
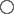
 Yes, in the clinic
-
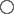
 Yes, on referral
-
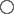
 No
-
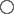
 Other

### []

Is a viral load test available for patients in your clinic?

### *

Please choose only one of the following:

-
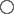
 Yes, in the clinic
-
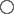
 Yes, on referral
-
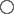
 No
-
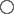
 Other

### []

What is the frequency of doing CD4 count for patients in care in your center

### *

Please choose only one of the following:

-
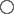
 Monthly
-
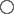
 Each 3 months
-
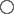
 Each 6 months
-
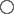
 Each year
-
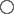
 Other

### []

What is the frequency of doing viral load testing for patients in your clinic?

### *

Please choose only one of the following:

-
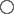
 Monthly
-
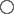
 Each 3 months
-
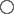
 Each 6 months
-
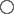
 Each year
-
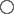
 Other

### []

Which drugs are available free of charge for patients in your clinic?

### *

Please choose all that apply:

-
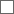
 ARV
-
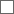
 Cotrim
-
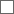
Other:

### []If free ARV drugs are available in your clinic, please tick which ones are free

Please choose all that apply:

-
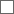
 AZT (zidovudine, Retrovir)
-
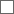
 TDF (tenofovir disoproxil fumarate)
-
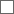
 FTC (emtricitabine )
-
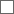
 3TC (lamivudine)
-
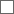
 NVP (nevirapine)
-
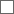
 IDV (indinavir)
-
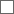
 RTV (ritonavir )
-
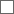
 d4T (stavudine)
-
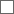
 ABC (abacavir)
-
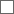
 LPV (lopinavir)
-
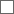
 ATV (atazanavir)
-
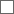
 Cotrimoxazole
-
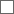
Other:

### []

What policy is in use in your clinic about starting treatment:

### *

Please choose only one of the following:

-
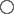
 Commencement when CD4 count < 200 cells /ml
-
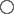
 Commencement when CD4 count < 350 cells /ml
-
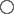
 Commencement when CD4 count < 500 cells /ml
-
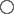
 Immediate commencement irrespective of CD4 count
-
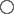
 Other

### []

What system is used for recording patient data in patient records?

### *

Please choose only one of the following:

-
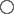
 Paper
-
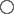
 Electronic
-
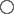
 Both
-
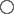
 Other

### []

Is there any system to aggregate and report patient data for the facility as a whole?

### *

Please choose only one of the following:

-
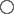
 Yes, it is performed by clinic staff
-
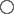
 Yes, it is sent elsewhere
-
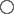
 No
-
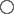
 Other

### []

Do you have referral mechanisms to other healthcare or diagnostic facilities?

### *

Please choose only one of the following:

-
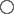
 Yes
-
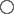
 No
-
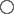
 Other

### []If you are able to refer patients, please list the types of referral facility you have available

Please write your answer here:
