# Supplementary material for: HIV treatment and monitoring patterns in routine practice: a multi-country retrospective chart review of patient care
Source: F1000Res. 2019 Jan 4;7:713. Originally published 2018 Jun 8. [Version 3] doi: 10.12688/f1000research.15169.3 (PMC6317496; doi:10.12688/f1000research.15169.3)
Supplement: Supplementary file 2 [file f1000research-7-19421-s0001.tgz › 702257a0-5761-487a-8f73-b84c2571c68a.doc]

HIV care in routine practice: protocol for a multi-country Peoples-uni descriptive study of treatments and tests on patients diagnosed HIV positive

*Background.*

While the optimal treatment for HIV may change as new evidence comes to light, best practice may require access to various aspects of care, clinical, immunological and virological information on patients at diagnosis and on follow-up, and access to appropriate drug choices. Clinical information might include important co-infections and/or co-morbidities, measures of adherence to therapy as well as genotypic resistance testing. In resource-limited settings, standardised regimens for the testing and treatment of patients with HIV may be implemented, without access to many of the above requirements for evidence-based best practice. While a number of reports are available from resource-limited settings, these are mainly in the context of patients enrolled in research centres or in research studies. We propose a study to explore the testing, treatment, care and follow-up among patients diagnosed with HIV in routine care settings in low- to middle-income countries where graduates of Peoples-uni work..

There are two parts to this study – information on what is available in the health care settings themselves, and information from patient records on the treatments and testing to which they have been subjected.

*Research questions.*

1. Among a group of patients diagnosed with HIV by a health care facility newly diagnosed between 2 and 3 years previously, what proportion have standards of care in terms of the tests and treatments they receive and follow up to 2 years after diagnosis documented in their records?
2. Among the health care facilities in which the above patients are cared, what treatment, testing and referral facilities are available?
3. What is the extent of the variation in the above measures between facilities and countries, as well as in the availability of appropriate evidence based practice guidelines?

*Study type*

This will be a retrospective cohort, records based study

*Study sample*

The reference population is the generality of patients with a new diagnosis of HIV, the source population is the patients presenting to a series of identified health care facilities, and the study sample is those patients presenting to the identified health care facilities with a new diagnosis of HIV in a six month period between January and June, 2013.

*Measurement of Study factors*

1) Data on individual patients from patient records: data on age, gender, and baseline clinical data will be collected as per the attached proforma at diagnosis, and at the 1- and 2-year follow-up periods.
2) Description of the system: Each centre will record the characteristics of the setting – country and city, hospital or other healthcare facility, number of patients seen, system of recording patient data in patient records and any system to aggregate and report patient data for the facility as a whole, private or public. What diagnostic, treatment, referral and follow-up facilities are available?

*Measurement of Outcome factors*

Details of the tests ordered and their results, and treatments ordered will be examined in the initial diagnostic phase, and up to two years after diagnosis, and recorded on a spreadsheet. If there are no data at the follow up periods, attempts will be made to contact the referring doctor for information. Individual patients will not be contacted.

Measurements will be made by the investigator, or a research assistant, following a pilot where the spreadsheet will be tested for feasibility in each setting. The spreadsheet will be piloted initially in one or two settings and adapted as required.

*Ethics requirements*

This will be a retrospective patient records study, consistent with an audit, and permission will not be requested from individual patients. However, considerable care will be taken not to reveal the identity of any individual. The spreadsheet for the recording of data will only have an identification number, and the key to the identity of each patient will be kept separately from the proformas, in a locked cabinet. Reporting of results will not identify any individual patient, nor the health care facility from where the patients are drawn.

Ethical permission to perform the study will be requested from each of the ethics committees of the relevant organisations taking part in the study, if required by the organisation.

*Statistical considerations*

This is a descriptive study, and sample size is not feasible to be determined in advance. Each centre is expected to obtain information from the first 100 patients presenting over a 6-month period, or from at least 100 patients.

Two data collection instruments have been developed, to allow data to be entered into a spreadsheet for descriptive analysis. One is for information about the clinic and availability of tests and treatment – facilities survey – and one is for information on patients – patient survey.

*Organisation*

The study is to be performed among alumni of the People's Open Access Education Initiative (Peoples-uni) and their colleagues and contacts. A steering group comprises:

Dr Baba Musa, Physician and Senior Lecturer, Aminu Kano Teaching Hospital, Kano, Nigeria

Mr Ado Abubakar, Senior Program officer, Institute of Human Virology, Abuja, Nigeria

Mr Everistus Ibekwe, Clinical Laboratory Scientist, State House Medical Center, Abuja, Nigeria

Professor Richard Heller, Coordinator, Peoples-uni, Emeritus Professor University of Manchester

Statistical advice is offered by two statisticians from Statistics Without Borders.

Alumni in Nigeria, Tanzania, Ghana, Uganda, Zambia, and Zimbabwe have agreed to participate.

*Funding*

No funding has been sought.
