# Supplementary material for: HIV treatment and monitoring patterns in routine practice: a multi-country retrospective chart review of patient care
Source: F1000Res. 2019 Jan 4;7:713. Originally published 2018 Jun 8. [Version 3] doi: 10.12688/f1000research.15169.3 (PMC6317496; doi:10.12688/f1000research.15169.3)
Supplement: Supplementary file 3 [file f1000research-7-19421-s0002.tgz › 46a458e2-e4c7-4f5a-83ff-124576688038.doc]

Coding sheet

Notes:

Missing data: use a . to indicate missing data, do not leave blank

Do not use , in any column

Do not add letters in data columns – eg height 180cm code 180 only

Repeat on each sheet

Column A: Site code. Start with letters eg Kano KA1

Column B: Patient ID. Start with a number eg 001

Sheet 1: Demographics

Column C: Date of first visit for diagnosis. Day/month/year as eg 01/03/2014

Column D: Date of Birth. Day/month/year as eg 01/03/1984 (if missing, code .)

Column E: Year of Birth (if date of birth not known). Eg 1984. Can be derived from age

Column F: Gender. Male =1 Female =2

Column G: Height in cm at first visit. Only code number. Round to nearest whole cm

Column H: Weight in Kg at first visit. Only code number. Round to nearest whole Kg

Column I: Mode of infection. Heterosexual =1; Homosexual =2 ; IV drug =3; Other =4

Column J: Mode of diagnosis. Blood test =1; AIDS defining illness =2

Column K: History of TB. Date day/month/year as eg 01/03/2014 or . If missing

Column L: History of Hepatitis B. Date day/month/year as eg 01/03/2014 or . If missing

Column M: History of Hepatitis C. Date day/month/year as eg 01/03/2014 or . If missing

Column N: Date of last visit found in records. Date day/month/year as eg 01/03/2014 or . If missing

Column O: Date of death if recorded. Date day/month/year as eg 01/03/2014 or . If missing

Sheet 2: CD4 count

Column C: Date of test Day/month/year as eg 01/03/2014

Column D: CD4 count as cells/µL Only include number as whole number Only use . For missing data

Sheet 3: Viral load

Column C: Date of test Day/month/year as eg 01/03/2014

Column D: HIV-RNA viral load (copies/mL) Code <50 =1; 50-999 =2; 1000-9999; 10000-99999 =3; > 100000 =4 Use . For missing data

Sheet 4: Illness

Column C: Date (at diagnosis or follow up) Day/month/year as eg 01/03/2014

Column D: Any AIDS Defining Illness Yes=1; No=2

Column E: Diagnosis. Code as follows (leave blank if no there is no AIDS defining illness):

1. Candidiasis of bronchi, trachea, or lungs
2. Candidiasis of esophagus
3. Cervical cancer, invasive
4. Coccidioidomycosis, disseminated or extrapulmonary
5. Cryptococcosis, extrapulmonary
6. Cryptosporidiosis, chronic intestinal (>1 month's duration)
7. Cytomegalovirus disease (other than liver, spleen, or nodes), onset at age >1 month
8. Cytomegalovirus retinitis (with loss of vision)
9. Encephalopathy, HIV related
10. Herpes simplex: chronic ulcers (>1 month's duration) or bronchitis, pneumonitis, or esophagitis (onset at age >1 month)
11. Histoplasmosis, disseminated or extrapulmonary
12. Isosporiasis, chronic intestinal (>1 month's duration)
13. Kaposi sarcoma
14. Lymphoid interstitial pneumonia or pulmonary lymphoid hyperplasia complex
15. Lymphoma, Burkitt (or equivalent term)
16. Lymphoma, immunoblastic (or equivalent term)
17. Lymphoma, primary, of brain
18. *Mycobacterium avium* complex or *Mycobacterium kansasii,* disseminated or extrapulmonary
19. *Mycobacterium tuberculosis* of any site, pulmonary, disseminated, or extrapulmonary
20. *Mycobacterium*, other species or unidentified species, disseminated or extrapulmonary
21. *Pneumocystis* *jirovecii* pneumonia
22. Pneumonia, recurrent
23. Progressive multifocal leukoencephalopathy
24. *Salmonella* septicemia, recurrent
25. Toxoplasmosis of brain, onset at age >1 month
26. Wasting syndrome attributed to HIV

Sheet 5: ARV treatment

Column C: Start date Day/month/year as eg 01/03/2014

Column D: End date Day/month/year as eg 01/03/2014

Columns E to R: individual ARV treatments. Start a new row for each new treatment and code 1 in the column for that drug, and give the date of starting in column C, and in column D the date where it has been stopped

Column R: Change reason. If a drug has been stopped, give the reason for the change: Code: 1= toxicity; 2= CD4 or viral load; 3= complication; 4=unknown

Column S: Any extra comment on the change reason
