# Supplementary material for: HIV treatment and monitoring patterns in routine practice: a multi-country retrospective chart review of patient care
Source: F1000Res. 2019 Jan 4;7:713. Originally published 2018 Jun 8. [Version 3] doi: 10.12688/f1000research.15169.3 (PMC6317496; doi:10.12688/f1000research.15169.3)
Supplement: Peer review history

# Peer review: Referee response for version 2

**DOI:** 10.5256/f1000research.17926.r42116

**Published:** 2018-12-31

**Authors:** Samuel A. Olowookere

**Affiliations:** 1 Department of Community Health, Obafemi Awolowo University, Ile-Ife, Nigeria

1. Methods: Kindly include the research question being answered. Study design needs to be made clearer in this section.
2. Measurement of study factors: Kindly specify important co-infections and co-morbidities.
3. Sample size consideration/selection: how were the centres selected? It is not clear how the size of 100 patients was arrived at. If the sample size and record selection was arbitrary this should be stated as a limitation of the study. What is standardised data collection? If this study is looking for variations, what of the similarities? The method section should give more background information on the centres chosen. This will include the health care worker/patient ratio, patient load and type of care provided to the patients.
4. What are the effects of missing values on the study findings? Efforts should be made by the authors to highlight reasons for missing values. A statement on the best way to address the missing values should be stated. What is the new information that this study added to the literature?

I have read this submission. I believe that I have an appropriate level of expertise to confirm that it is of an acceptable scientific standard, however I have significant reservations, as outlined above.

# Response

**Published:** 2019-01-02

**Authors:** Richard Heller

**Affiliations:** People's Open Access Education Initiative (Peoples-uni), Australia

Comments from Referee 3, and how we have responded and made changes ( *Referee comments in italics*).

*Methods: Kindly include the research question being answered. Study design needs to be made clearer in this section.*

Response: Paragraph 1 in the Methods section has been revised as follows: “Interest in participating in this study was sought among the Masters of Public Health Alumni of Peoples-uni. A protocol was developed by those who responded ( Supplementary File 1) which included the Research questions as follows: 1. Among a group of patients diagnosed with HIV by a health care facility newly diagnosed between 2 and 3 years previously, what proportion have standards of care in terms of the tests and treatments they receive and follow up to 2 years after diagnosis documented in their records? 2. Among the health care facilities in which the above patients are cared, what treatment, testing and referral facilities are available? 3. What is the extent of the variation in the above measures between facilities and countries, as well as in the availability of appropriate evidence based practice guidelines? A retrospective patient cohort was created via standardized record review, and clinic characteristics determined through an online survey form.”

*Measurement of study factors: Kindly specify important co-infections and co-morbidities.*

Response: We have changed the sentence in the section of Measurement of study factors from ‘Clinical information included clinical, immunological and virological information on patients at diagnosis and on follow-up, prescription of ART and other drug choices, important co-infections and/or co-morbidities.’ to the following: “Clinical information included clinical, immunological and virological information on patients at diagnosis and on follow-up, prescription of ART and other drug choices. Potentially important co-infections and/or co-morbidities, identified from the literature, were coded as indicated in Supplementary File 2,” The list is long and since the file is easily accessible to the reader we think that this is a reasonable way to meet the request of the Referee while not adding too much to the size of the paper.

*Sample size consideration/selection: how were the centres selected? It is not clear how the size of 100 patients was arrived at. If the sample size and record selection was arbitrary this should be stated as a limitation of the study. What is standardised data collection? If this study is looking for variations, what of the similarities? The method section should give more background information on the centres chosen. This will include the health care worker/patient ratio, patient load and type of care provided to the patients.*

Response: As indicated in the paper (Methods) ‘Interest in participating in this study was sought among the Masters of Public Health Alumni of Peoples-uni’ and (Results) ‘Ten alumni expressed interest in participating, three centres were able to pilot the data collection instrument and four centres in three countries participated in the data collection for the study.’ We have added to the sentence in the Methods section as follows: “Interest in participating in this study was sought among the Masters of Public Health Alumni of Peoples-uni. There was no prior determination of the numbers of centres required or their geographical setting.” We have added to the section on study limitations as follows: “Since this is a descriptive study and not hypothesis testing, it was not feasible to determine sample size in advance. A pragmatic approach was adopted, with each centre expected to obtain information from the first 100 patients presenting over a 6-month period, or from at least 100 patients.”

We have changed the first sentence in the Discussion to the following: “Data were collected according to the standardised data collection instrument ( Supplementary File 2), with data on treatment regimes and CD4 counts relating to the latest information in the records (dates mainly between 2015 and 2017).”

We believe that the background information that the Referee requires on the centres chosen is fully reported in Table 1 in the Results section.

*What are the effects of missing values on the study findings? Efforts should be made by the authors to highlight reasons for missing values. A statement on the best way to address the missing values should be stated. What is the new information that this study added to the literature?*

Response: Unfortunately, we do not have information on whether missing values are due to the tests not having been performed, or the information not having been recorded. As we have mentioned in the Discussion section ‘Some of the absence of viral load might be poor record keeping, and the high proportion of undetectable results might reflect an absent rather than a low test result, and hence not an indicator of treatment success.’ and in the section on study limitations ‘We have found missing data where it should ordinarily not be missing. We cannot comment on other factors that may have contributed to missing data, such as whether tests were not done or if they were not recorded.’ We have added the following in the study limitations section in response to the Referee’s request: “A future study should perform an audit of the medical records to determine the reason for missing data and hence the potential effect on the study findings.”

In response to the Referee’s request to identify the new information added by our study, we have added the following sentences to the Discussion section (paragraph 7) to follow our original sentence ‘Here we present combined data on health system related measures across multiple sub-Saharan African HIV treatment sites.’ the additional sentences read: “Hence, this study adds to the literature a current examination of the routine care provided to patients with HIV, rather than that in the context of a clinical trial. The study adds information from a number of centres in multiple countries, using a common protocol, of both the characteristics of the clinics and of the care given in these clinics.”

# Peer review: Referee response for version 2

**DOI:** 10.5256/f1000research.17926.r38719

**Published:** 2018-10-12

**Authors:** Thomas Crellen

**Affiliations:** 1 Mahidol Oxford Tropical Medicine Research Unit (MORU), Bangkok, Thailand

The authors have corrected minor issues in the article, though I retain my reservations around the study design and the lack of a research question.

I have read this submission. I believe that I have an appropriate level of expertise to confirm that it is of an acceptable scientific standard, however I have significant reservations, as outlined above.

# Response

**Published:** 2018-10-12

**Authors:** Richard Heller

**Affiliations:** People's Open Access Education Initiative (Peoples-uni), Australia

I am sorry that you did not make your reservations clear in your first report. With regard to the research question, we stated in the Introduction "This study was designed to explore variation in the evaluation, treatment, care and follow-up among patients diagnosed with HIV in routine care settings in low- to middle-income countries". With regard to the study design, we stated in the Discussion "Data were collected in a standardised way, with data on treatment regimes and CD4 counts relating to the latest information in the records (dates mainly between 2015 and 2017). While we initially planned to collect data on a cohort of patients enrolled two years before data collection in each clinic, this did not prove feasible and patients had presented to the clinics at variable times."

# Peer review: Referee response for version 2

**DOI:** 10.5256/f1000research.17926.r38718

**Published:** 2018-10-11

**Authors:** Sehlulekile Gumede-Moyo

**Affiliations:** 1 Department of Population Health, Faculty of Epidemiology and Population Health, London School of Hygiene and Tropical Medicine, London, UK

There has been some improvements in the current draft and most of the issues raised have been addressed. However l was wondering whether it’s possible for the authors to comment to the patient doctor ratio with regards to co-trimoxazole distribution.

I have read this submission. I believe that I have an appropriate level of expertise to confirm that it is of an acceptable scientific standard.

# Response

**Published:** 2019-01-02

**Authors:** Richard Heller

**Affiliations:** People's Open Access Education Initiative (Peoples-uni), Australia

Thank you for your comments. We could not demonstrate an obvious relationship between the doctor/patient ratio and use of co-trimoxazole, although it is an intriguing suggestion. A study including a larger sample of clinics might be able to explore this further.

# Peer review: Referee response for version 1

**DOI:** 10.5256/f1000research.16524.r37307

**Published:** 2018-09-17

**Authors:** Sehlulekile Gumede-Moyo

**Affiliations:** 1 Department of Population Health, Faculty of Epidemiology and Population Health, London School of Hygiene and Tropical Medicine, London, UK

The paper is well written, however in some instances the authors have used outdated references. In the case of Nigeria where there are 2 health facilities with different caseloads per month, the authors could also enlighten us on the impact of having a high and low case load as presented.

 The authors should also enlighten us on whether the records indicate the dates when the CD4 count and viral load tests were taken and when they were reported back. I suppose this of great importance since, there are reports of patients who have their blood samples taken and never receive results.

 1) Is The study design appropriate and is the work technically sound?

 The study design wand the work is technically, as the authors endeavoured to analyse multi-country settings

 2. Are sufficient details of methods and analysis provided to allow replication by others?

 The methods and analysis were sufficient, however in the case of Nigeria where there are 2 health facilities with different case loads per month; the authors could also enlighten us on the impact of having a high and low case load as presented.

 3. If applicable, is the statistical analysis and its interpretation appropriate?

 The statistical analysis was appropriate, although the authors should also enlighten us on whether the records indicate the dates when the CD4 count and viral load tests were taken and when they were reported back. I suppose this of great importance since, there are reports of patients who have their blood samples taken and never receive results.

 4. Are the conclusions drawn adequately supported by the results?

 The authors however need to address the issues that have been raised above.

I have read this submission. I believe that I have an appropriate level of expertise to confirm that it is of an acceptable scientific standard, however I have significant reservations, as outlined above.

# Response

**Published:** 2018-09-22

**Authors:** Richard Heller

**Affiliations:** People's Open Access Education Initiative (Peoples-uni), Australia

Thank you for your generally positive comments. In the revised version submitted, we have added four recent references, and given further details on the dates of CD4 counts and viral load tests (although we have no data on the time that the results were reported to the patients). We have also discussed the reasons that the two Nigerian clinics might have different caseloads and the potential impact of this.

# Peer review: Referee response for version 1

**DOI:** 10.5256/f1000research.16524.r37430

**Published:** 2018-09-03

**Authors:** Thomas Crellen

**Affiliations:** 1 Mahidol Oxford Tropical Medicine Research Unit (MORU), Bangkok, Thailand

This article by Musa and colleagues uses a snapshot of patient records from four HIV clinics in Nigeria, Zambia and Uganda to describe the variability in a number of patient measures. Data are not reported longitudinally and all analysis is descriptive.

 The most useful part of this report is to show that ordinary clinics in countries with some of the highest HIV burdens globally do not confirm with WHO guidelines. For instance, only commencing ART when CD4 cells <350/ul. Further, the high amount of missing data in viral loads suggest there are logistical or cost challenges to routine testing.  

 It is perhaps unsurprising that clinics in different countries vary in their patient characteristics and levels of reporting. The variability in CD4 counts is not particularly informative as it is not linked to length of time under ART. No research questions are addressed.

 Table 2 should be amended as some percentage values are missing and figures are presented to different numbers of significant figures.

 Despite the descriptive nature of the study, the authors are to be commended on presenting hard to access data from routine health care settings in sub-Saharan Africa. I hope that they will be able to take longitudinal data in the future and better understand how clinical practice in these settings is linked to patient outcomes.

I have read this submission. I believe that I have an appropriate level of expertise to confirm that it is of an acceptable scientific standard, however I have significant reservations, as outlined above.

# Response

**Published:** 2018-09-22

**Authors:** Richard Heller

**Affiliations:** People's Open Access Education Initiative (Peoples-uni), Australia

Thank you for your positive comments on the paper. In the revised version submitted, we have tried to emphasise the points you make, and have amended Table 2 as requested.

# Response

**Published:** 2019-01-02

**Authors:** Richard Heller

**Affiliations:** People's Open Access Education Initiative (Peoples-uni), Australia

In a later revision of the paper, paragraph 1 in the Methods section has been revised as follows to include the research question: “Interest in participating in this study was sought among the Masters of Public Health Alumni of Peoples-uni. A protocol was developed by those who responded ( Supplementary File 1) which included the Research questions as follows: 1. Among a group of patients diagnosed with HIV by a health care facility newly diagnosed between 2 and 3 years previously, what proportion have standards of care in terms of the tests and treatments they receive and follow up to 2 years after diagnosis documented in their records? 2. Among the health care facilities in which the above patients are cared, what treatment, testing and referral facilities are available? 3. What is the extent of the variation in the above measures between facilities and countries, as well as in the availability of appropriate evidence based practice guidelines? A retrospective patient cohort was created via standardized record review, and clinic characteristics determined through an online survey form.”
